# Dynamic multilayer functional connectivity detects preclinical and clinical Alzheimer’s disease

**DOI:** 10.1093/cercor/bhad542

**Published:** 2024-01-11

**Authors:** Anna Canal-Garcia, Dániel Veréb, Mite Mijalkov, Eric Westman, Giovanni Volpe, Joana B Pereira, For the Alzheimer’s Disease Neuroimaging Initiative

**Affiliations:** Department of Clinical Neuroscience, Karolinska Institutet, Stockholm 17165, Sweden; Department of Clinical Neuroscience, Karolinska Institutet, Stockholm 17165, Sweden; Department of Clinical Neuroscience, Karolinska Institutet, Stockholm 17165, Sweden; Department of Neurobiology, Care Sciences and Society, Karolinska Institutet, Stockholm 17165, Sweden; Department of Physics, University of Gothenburg, Gothenburg 40530, Sweden; Department of Clinical Neuroscience, Karolinska Institutet, Stockholm 17165, Sweden

**Keywords:** AD, cognition, pathology, rs-fMRI, temporal brain networks

## Abstract

Increasing evidence suggests that patients with Alzheimer’s disease present alterations in functional connectivity but previous results have not always been consistent. One of the reasons that may account for this inconsistency is the lack of consideration of temporal dynamics. To address this limitation, here we studied the dynamic modular organization on resting-state functional magnetic resonance imaging across different stages of Alzheimer’s disease using a novel multilayer brain network approach. Participants from preclinical and clinical Alzheimer’s disease stages were included. Temporal multilayer networks were used to assess time-varying modular organization. Logistic regression models were employed for disease stage discrimination, and partial least squares analyses examined associations between dynamic measures with cognition and pathology. Temporal multilayer functional measures distinguished all groups, particularly preclinical stages, overcoming the discriminatory power of risk factors such as age, sex, and APOE ϵ4 carriership. Dynamic multilayer functional measures exhibited strong associations with cognition as well as amyloid and tau pathology. Dynamic multilayer functional connectivity shows promise as a functional imaging biomarker for both early- and late-stage Alzheimer’s disease diagnosis.

## Introduction

Functional magnetic resonance imaging (fMRI) is a non-invasive neuroimaging technique that measures changes in blood oxygenation in response to neural activity (Ogawa et al. 1990; Heeger and Ress 2002; Logothetis 2003). Although this technique has shown great promise in identifying early functional changes in Alzheimer’s disease (AD), its role in the diagnosis of different AD stages is currently unclear. This is due to the fact that most results reported by previous studies have been inconsistent (Schultz et al. 2017; Sepulcre et al. 2017; Corriveau-Lecavalier et al. 2020), with some showing alterations in functional connectivity (FC) on resting-state fMRI (rs-fMRI) after fibrillar amyloid deposition (Mormino et al. 2009; Sperling et al. 2009), and others showing changes before amyloid accumulation (Sheline et al. 2010). Moreover, the diagnostic accuracy of fMRI has been questioned due to its high variability across individuals (Biswal et al. 1995). Thus, although fMRI has the potential to provide important insights into the pathophysiology of AD, further research is needed to determine its clinical utility in the diagnosis and management of this disorder.

Until now, most rs-fMRI connectivity studies performed in AD assumed that FC patterns in the brain remain stable during the entire rs-fMRI scanning (Supekar et al. 2008; Sperling et al. 2009; Sanz-Arigita et al. 2010). However, it is now generally accepted that changes in spontaneous fluctuations and correlations among various brain regions are far from being static, but change dynamically over time, even when the brain is at rest (Hutchison et al. 2013; Demirtaş et al. 2017; Park et al. 2017; Wu et al. 2022). However, conventional approaches are not sensitive to these dynamic connectivity abnormalities in AD because they do not take into account temporal dynamics.

Recent studies of FC in AD have expanded to include time-varying FC as opposed to the conventional statis FC (time-averaged FC). Dynamic FC have been shown to provide a superior predictive accuracy in distinguishing between healthy individuals and individuals with mild cognitive impairment (MCI) or AD compared to static FC measures (Chen et al. 2016; Wee et al. 2016; Chen et al. 2017; de Vos et al. 2018). Therefore, analyzing the topological characteristics of dynamic FC networks may be more effective and robust in revealing AD-related connectivity alterations (Preti et al. 2017).

Novel methods in network neuroscience, such as multilayer network modeling and multilayer community detection (Mucha et al. 2010), have been used to analyze brain activity over time measured with fMRI in healthy individuals (Pedersen et al. 2018; Malagurski et al. 2020; Puxeddu et al. 2020) and in patients with schizophrenia (Gifford et al. 2020; Yang et al. 2022). This approach normally separates fMRI data into a number of time windows or snapshots and a correlation measure is used to build a functional network for each time window (De Domenico 2017). As a result, a time-varying functional multilayer network can be constructed, where each layer encodes a functional snapshot of brain activity. In comparison to conventional approaches, this methodology is richer because it can provide measures of functional changes over the scanning period, such as network flexibility, which describes how frequently different brain regions switch from a module to another one over time (Bassett et al. 2011). To further investigate this dynamic organization, the co-activation of regions (or resting-state networks, RSNs) in communities can be represented by the module allegiance matrix, where pairs of brain regions that often co-activate in the same community or module across time have high allegiance values. Based on this, other measures of dynamic community organization can be computed, such as the dynamic recruitment coefficient, which is the likelihood that the regions of an RSN remain within the same network over time, and the dynamic integration coefficient, which is the likelihood that the regions of an RSN are assigned to communities from other RSNs (Mattar et al. 2015).

While there are several dynamic FC studies in patients with neurodegenerative disorders such as AD (de Vos et al. 2018; Arbabyazd et al. 2023; Sendi et al. 2023), to our knowledge, none have employed the novel methodology of multilayer dynamic functional connectivity. Since rs-fMRI is a non-invasive imaging modality that could be used in clinical practice without posing an additional burden on patients, the aim of our study is to evaluate the clinical value of the dynamic modular organization across different stages of AD, including cognitively normal (CN) individuals, patients with MCI and patients with AD dementia all with Aβ pathology (Aβ+) in comparison to CN without Aβ pathology (Aβ−). To achieve this, we analyzed rs-fMRI data using a dynamic approach and examined the role of flexibility, dynamic integration, and dynamic recruitment within RSNs between groups and assessed their relationship with pathology and cognition across the AD continuum. We hypothesized the temporal dynamics from the rs-fMRI scans could reliably identify different AD stages that could be used for their diagnosis and could be incorporated into a more comprehensive and detailed AD amyloid cascade model.

## Materials and methods

### Participants

The data used in this study were obtained from the Alzheimer’s Disease Neuroimaging Initiative 3 (http://adni.loni.usc.edu). We included subjects with T1-weighted and functional MRI data that passed quality control before and after image preprocessing. In addition, all included subjects had amyloid-PET (^18^F-Florbetapir) and tau-PET (^18^F-Flortaucipir) scans in addition to demographic and clinical data.

The ADNI was launched in 2003 as a public-private partnership, led by Principal Investigator Michael W. Weiner, MD. The primary goal of ADNI has been to test whether serial MRI, PET, other biological markers, and clinical and neuropsychological assessment can be combined to measure the progression of MCI and early AD. The inclusion/exclusion criteria from ADNI can be found at http://www.adni-info.org/. In brief, all participants were between the ages of 55 and 90 years, had completed at least 6 years of education, and were fluent in Spanish or English. Control subjects had Mini-Mental State Examination (MMSE) scores between 24 and 30, a Clinical Dementia Rating-Sum of Boxes (CDR-SB) score of 0, and lacked depression, MCI, or dementia. Inclusion criteria for the MCI group followed the Peterson criteria (Petersen et al. 1999) for amnestic MCI. AD participants met the National Institute for Neurological and Communicative Disorders and Stroke-Alzheimer’s Disease and Related Disorder Association (NINDS/ADRDA) criteria for probable AD, had an MMSE score between 18 and 26, and a CDR-SB of 0.5–1.0. Exclusion criteria for all participants comprised history of structural brain lesions or head trauma, significant neurological disease other than incipient AD, and the use of psychotropic medications that could affect memory. Finally, APOE ϵ4 genotyping was carried out and registered in the ADNI database at the time of participant enrollment. Specifically, DNA that was isolated by Cogenics from a 3-mL sample of EDTA blood was used to genotype the two SNPs (rs429358, rs7412) that characterize the epsilon 2, 3, and 4 alleles.

The ADNI is conducted in accordance with the ethical standards of the institutional research committees and with the 1975 Helsinki declaration and its later amendments. Written informed consent, obtained from all subjects and/or authorized representatives and study partners, and ethical permits have been obtained at each participating site of ADNI and we have signed the data user agreements to analyze the data.

### Image acquisition

All participants underwent 3 T MRI using T1-weighted and resting-state fMRI. T1-weighted imaging was performed using a sagittal 3D accelerated MPRAGE sequence with full head coverage, voxel size = 1 × 1 × 1 mm^3^, field of view = 208 × 240 × 256 mm^3^, repetition time = 2300 ms, and inversion time = 900 ms. fMRI was conducted using an axial echo planar imaging sequence with voxel size = 3.4 × 3.4 × 3.4 mm^3^, field of view = 220 × 220 × 163 mm^3^, duration of 10 min, 200 volumes, repetition time = 3000 ms, echo time = 30 ms, and flip angle = 90°. All subjects underwent positron emission tomography (PET). ^18^F-florbetapir PET images were acquired in four 5-min frames, 50–70 min after injection of approximately 10 mCi. Then, the 4 frames were coregistered, averaged and interpolated to a uniform image and voxel size (160 × 106 × 96 voxels, 1.5 mm^3^). Finally, ^18^F-flortaucipir PET images were acquired following an injection of 10.0 ± 1.0 mCi dose of [^18^F]-AV1451. They were acquired for 30 min in six frames (5 min per frame), 75–105 min after the injection. More information about the MRI and PET acquisition methods is provided at https://adni.loni.usc.edu/data-samples/data-types/.

### Image preprocessing

Functional and structural MRI scans were pre-processed using fMRIPrep (v20.2.4) (Esteban et al. 2019). The first two volumes of the functional scans were removed to account for steady-state magnetization effects. Then, functional images were motion-corrected, adjusted for slice timing effects, skull-stripped, and co-registered to a 2 mm resolution MNI152 standard template space. The two-stage registration approach was performed with Freesurfer (Fischl et al. 1999) and ANTs (Avants et al. 2014). To further account for motion effects and remove confounding signals from the white matter and cerebrospinal fluid, nuisance regression using the 24-parameter head motion model (Friston et al. 1996) was employed. Finally, volumes underwent high-pass filtering with a cut-off of 0.01 Hz, a common frequency cut-off for infraslow resting state oscillations, which are typically considered to occur between 0.01 and 0.1 Hz (Woolrich et al. 2001; Picchioni et al. 2011). Regarding the PET scans, we used the standard uptake value ratios (SUVR) obtained from the preprocessed data provided by ADNI. A detailed description of the PET preprocessing methods can be found at: https://adni.loni.usc.edu/methods/pet-analysis-method/pet-analysis/. In summary, the 5-min PET frames were co-registered, averaged, and co-registered to the T1-weighted MRI images of each participant. Finally, normalized SUVR maps were created by using the cerebellar gray matter as a reference region (Johnson et al. 2016). For the purposes of our study, all subjects were classified into four groups according to clinical diagnosis and an Aβ PET SUVR threshold of 1.11 (cut-off established by ADNI) based on previous evidence showing that Aβ pathology is one of the earliest events in AD and is eventually followed by cognitive decline and dementia (Jack et al. 2013). After removing the outliers based on the results of the dynamic multilayer functional connectivity measures (see Section 2.6 about outliers’ removal), the groups consisted of 86 Aβ-negative CN, 37 Aβ-positive CN, 34 Aβ-positive MCI patients, and 22 Aβ-positive AD patients. Patients with MCI and AD without Aβ pathology were excluded since they are not part of the AD continuum and may potentially have a non-AD disorder (Jack et al. 2018).

### Network construction

**Fig. 1:**
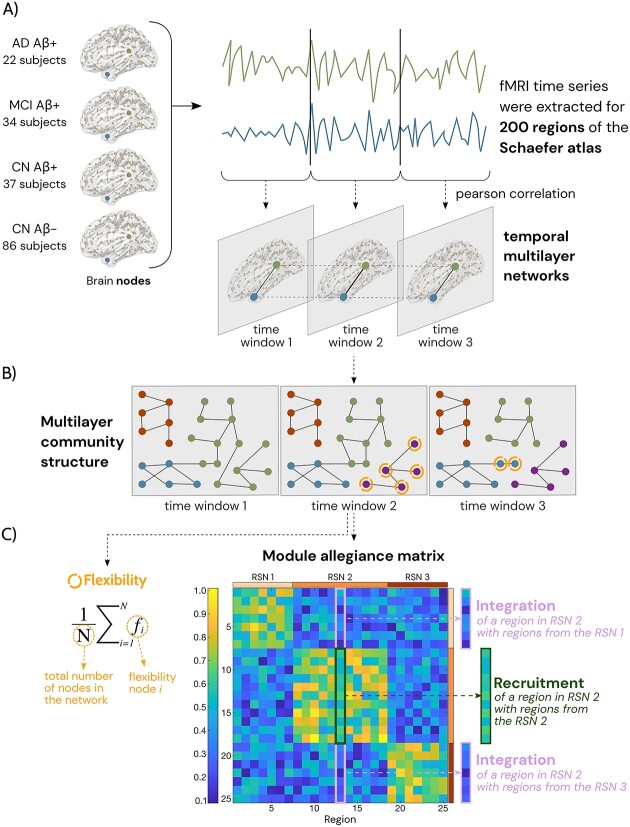
Visual representation of the methodology. From each group, we extracted the rs-fMRI time-series for 200 regions of the Schaefer atlas and then divided into 19 non-overlapping time windows of 30-s duration in order to obtain an adjacency matrix from each time window (A). Then we calculated the individual multiplex communities (B). Lastly, we evaluated the flexibility and calculated the module allegiance matrix, which represents the probability that two brain regions are part of the same community across the time windows (C). We also computed the dynamic recruitment coefficient and the dynamic integration coefficient of the different RSNs that summarize the results from the module allegiance (C).

To build the fMRI networks, 200 cortical regions from the Schaefer atlas (Schaefer et al. 2018) were used to define the nodes in our temporal multilayer networks (Fig. 1A). These regions can be grouped into seven well-known RSNs according to the Yeo-Krienen atlas (Thomas Yeo et al. 2011): the visual (VIS), somatomotor (SM), dorsal attention network (DAN), salience ventral attention network (SVAN), limbic (LIMB), control (CON), and default mode network (DMN). The time series from all the regions were divided into 19 non-overlapping time windows of 30 s (thus using 190 volumes of the fMRI scans), based on previous research suggesting time windows of 30–60 s for studying dynamic FC in fMRI (Leonardi and Van De Ville 2015; Gifford et al. 2020). Then, for each time window, we correlated the time series between each pair of nodes using Pearson’s correlations in order to build a time-varying functional multilayer network. This step was done for each group using the BRAPH 2 (Mijalkov et al. 2017; Gómez-Ruiz et al. 2022) software (Pipeline Functional Ordered-Multiplex Analysis using Weighted Undirected graphs). Thus, a temporal multilayer network was created for each participant consisting of 19 layers of 200 × 200 correlation matrices per layer with the diagonal and negative values set to zero to prevent self-connections. The layers in the resulting participant multilayer networks were connected in a consecutive or sequential order, and only connections between the corresponding brain regions were allowed. In a secondary analysis, we also assessed dynamic connectivity using two additional time windows: 5 windows of 120 s (using 195 time points) and 9 windows of 60 s (using 180 time points), which showed similar results in most of the RSNs, but were overall less sensitive in detecting changes across the AD continuum (Supplementary Fig. 1). In order to have time windows of equal size, the number of timepoints employed in each configuration differs.

### Multilayer network analysis

#### Multilayer community structure

Community detection methods such as the maximization of the modularity quality function (Newman and Girvan 2004) are frequently used to cluster the nodes in a network. Compared to a null model, communities represent groups of nodes that are more highly connected to one another than to nodes outside of their community (Newman 2006). In order to identify network organization in temporal multilayer networks (Fig. 1B), we used the generalized multilayer modularity (Mucha et al. 2010), calculated as follows: $Q=\frac{1}{2\mu}\sum_{ij s r}\left[\left({A}_{ij s}-\gamma\ {P}_{ij s}\right){\delta}_{sr}+{\delta}_{ij}{\omega}_{jsr}\right]\delta \left({g}_{is},{g}_{jr}\right)$,where μ is the total weights of the edges, ${A}_{ijs}$ is the adjacency matrix between nodes *i* and *j* at layer *s*, γ is the resolution parameter, which sets the weights of intralayer connections at layer *s*, ${P}_{ijs}$ is the associated null matrix (i.e. Newman–Girvan null model) at layer *s*, ${\delta}_{sr}$= 1 if *s* = *r* and 0 otherwise, ω is the temporal resolution parameter which determines the weights of the inter-layer edges, ${\delta}_{ij}$= 1 if *i* = *j* and 0 otherwise, ${g}_{is}$ and ${g}_{jr}$ are the community allegiances of node *i* at layer *s* and node *j* and layer *r* respectively, and $\delta \left({g}_{is},{g}_{jr}\right)$ = 1 if the community allegiances ${g}_{is}$ and ${g}_{jr}$ of nodes *i* and *j* at layer *s* and *r* are the same and 0 otherwise.

The null model matrix ${P}_{ijs}$ is obtained by randomizing the edges of each layer, while maintaining the layer node’s strength (Newman and Girvan 2004; Newman 2006). By varying the resolution parameter γ, we can control the size and number of the detected communities or modules. Low values of γ produce fewer but larger communities, while high values of γ produce more but smaller communities. The parameter ω controls the weights of the edges between layers. Small values of ω highlight the unique modular structure of each time window and may thus reflect community structures that are only present in a certain time window, while larger values of ω highlight the shared modular structure across time windows, representing potential community structures that do not change over time (Puxeddu et al. 2020). Since we were interested in intermediate regimes where communities can get reconfigured over time windows while still maintaining their reliability over consecutive time windows, we chose a temporal parameter ω = 0.5 with a resolution parameter γ = 1.

To optimize the multilayer modularity we adapted the code of the generalized version of the Louvain algorithm implemented in MATLAB provided by the genlouvain package (Jeub et al. 2011–2019). Using this algorithm, the multilayer communities are obtained by maximizing the multilayer modularity through several iterations until the most optimal and stable module partition is found. Since the multilayer maximization algorithm is stochastic, we calculated it for 100 repetitions. We kept the results for the 100 optimizations, where the resulting output at each repetition is a number for each node that indicates the community assignment in each layer. By using the multilayer community detection algorithm, the communities of each layer are comparable since they are obtained in the same modularity optimization.

#### Measures of dynamic community structure

To further analyze the dynamic temporal community structure, we employ measures such as the flexibility and the module allegiance matrix (Fig. 1C). The flexibility of a node describes how often a region changes of community allocation across successive temporal layers. This measure has also been described as node switching (Bassett et al. 2011; Pedersen et al. 2018). The module allegiance matrix is the likelihood of two brain regions belonging to the same community over time and repetitions. High allegiance values are observed in pairs of brain areas that frequently co-activate in the same community across time and optimizations. By plotting the module allegiance matrix, we can visually identify the dynamic roles of the well-known RSNs (Mattar et al. 2015), where the diagonal of the matrix represents the dynamic recruitment and the off-diagonal the dynamic integration (Bassett et al. 2015; Mattar et al. 2015). The dynamic recruitment coefficient measures the likelihood that regions of a RSN are constantly assigned to the same module over different time layers and repetitions, whereas the dynamic integration coefficient quantifies the likelihood of a region being assigned to the same module as regions from other RSNs across time layers and repetitions (Mattar et al. 2015). We used the definition and code functions from the Network Community Toolbox to calculate all these measures from the multilayer community structure (Network Community Toolbox (n.d)).

### Statistical analysis

To assess differences between groups (CN Aβ−, CN Aβ+, MCI Aβ+, AD Aβ+) in demographic, clinical and genetic variables, the Kruskal–Wallis rank sum test was applied due to the non-normal distribution of the data using R Studio (version 4.2.1).

To assess the statistical significance of the differences between groups in the network measures (flexibility, recruitment, and integration of the RSNs), we carried out nonparametric permutation tests with 10,000 replicates (Bassett et al. 2008; He et al. 2008). These comparisons were conducted after removing outliers, which were detected using the robust median absolute deviation (MAD) method (values 3 MAD away from the median are considered as outliers) on the average dynamic connectivity network measures. To control our results for multiple comparisons, we applied false discovery rate (FDR) corrections (*q* < 0.05; Benjamini and Hochberg 1995) across all group comparisons for the three dynamic measures in the different seven RSNs.

To assess the predictive power of our dynamic functional measures for group classification, we ran logistic regression models to discriminate the CN Aβ− group from each Aβ + group. In order to identify the best models based on the 21 variables (3 functional measures for 7 RSN), we employed a logistic stepwise model, which uses the Akaike information criterion (AIC) to select the best predictive variables. We ran three models for each group comparison: one with the dynamic functional measures (model F; Functional), the second with AD risk factors age, sex and APOE ϵ4 (model R; Risk factors), and the last one combining variables from the two previous models (model F + R). We employed the rocit R-package to compute the receiver operating characteristic (ROC) curves, and the cutpointr R function was utilized to derive AUC values using a bootstrap procedure with 1000 replicates, maximizing both sensitivity and specificity. Then the statistical difference between models was evaluated using Kruskal–Wallis tests applied to the 1000 AUC replicates.

Finally, we performed a partial least squares (PLS) regression analysis to assess how our dynamic functional measures (the same 21 variables we used in the logistic regression models) were associated with pathology and cognition across all Aβ positive subjects. We fit a PLS regression model for each response variable (global cognition, memory, tau-PET, and amyloid-PET) independently. We selected the MMSE as a measure of global cognition and the delayed word recall item of the Alzheimer’s Disease Assessment Scale–Cognitive Subscale (ADAS Q4) as a measure of memory. Tau pathology was assessed using partial volume corrected tau-PET SUVR in Braak stages I-IV (Schöll et al. 2016), whereas amyloid pathology was assessed using amyloid-PET SUVR in a global composite region (Landau et al. 2015). All PLS models additionally included age, sex, education, and the presence of cognitive impairment. Prior to applying the PLS algorithm, all data underwent log transformation to address skewness in some functional measures, followed by scaling (z-scores). The optimal number of components for the PLS analysis was determined in each case using cross-validation (Krämer and Braun 2007; Krämer and Sugiyama 2011) as well as by minimizing the mean squared error. The best number of latent variables (LVs) was as follows: global cognition = 5, memory = 2, tau-PET = 2, amyloid-PET = 3. The contribution of each variable to the prediction was assessed through variable importance in projection (VIP) scores. VIP scores were calculated as the summation of PLS weights across LVs, weighted by the variance explained by each LV. Variables were considered significant predictors if their VIP score exceeded 1 (Chong and Jun 2005).

## Results

The characteristics of the sample can be found in Table 1. Age, sex, education, APOE ϵ4, MMSE, Modified Preclinical Alzheimer Cognitive Composite (mPACCTrailsB), ADAS Q4, Rey’s Auditory Verbal Learning Test (RAVLT forgetting), tau-PET SUVR, and amyloid-PET SUVR were compared across all groups with the Kruskal–Wallis rank sum test. As expected, MCI and AD patients had worse memory and global cognition and there was a higher prevalence of the APOE ϵ4 allele in the Aβ-positive groups compared to the Aβ-negative group. Additionally, the Aβ-positive groups had an older age than the Aβ-negative group.

**Table 1:** Characteristics of the sample.

	CN A**β**− (*n* = 86)	CN A**β**+ (*n* = 37)	MCI A**β**+ (*n* = 34)	AD A**β**+ (*n* = 22)	*P*-value
Age (years)	75.12 (10.39)	78.39 (9.18)	79.59 (9)	82.39 (5.22)	<0.001
Sex (f/m)	49/37	17/20	17/17	10/12	0.611
Education (years)	18 (2)	16 (5)	16 (4)	16 (3.75)	0.092
APOE ϵ4 (%)	23.26	45.95	55.88	54.55	<0.001
MMSE	29 (1)	29 (2)	29 (2)	22 (7.5)	<0.001
mPACCTrailsB	1.37 (3.51)	−0.32 (3.99)	−4.08 (6.63)	−16.09 (9.04)	<0.001
ADAS Q4	2 (2)	2 (2)	5 (3)	8 (0)	<0.001
RAVLT forgetting	2 (4)	4 (4)	5 (3)	5 (3)	<0.001
Tau-PET (PVC) Braak I-IV SUVR	1.75 (0.22)	1.85 (0.28)	2.16 (0.66)	2.51 (0.96)	<0.001
Amyloid-PET Global SUVR	0.998 (0.08)	1.262 (0.26)	1.338 (0.27)	1.4 (0.269)	<0.001

Values represent medians for each group and are followed by the interquartile range in parenthesis, except for sex. Comparisons between groups were performed using Kruskal–Wallis tests for continuous variables and with Chi-squared tests for binary variables. CN, cognitively normal; MCI, mild cognitive impairment; AD, Alzheimer’s disease; Aβ, amyloid-β; MMSE, Mini-Mental State Examination; mPACC, Modified Preclinical Alzheimer Cognitive Composite; ADAS Q4, delayed word recall item of the Alzheimer’s Disease Assessment Scale–Cognitive Subscale; RAVLT, Rey’s Auditory Verbal Learning Test; PET, positron emission tomography; PVC, partial volume corrected; SUVR, standardized uptake value ratio.

The average module allegiance matrices for each group in Supplementary Fig. 2 provide an overview of how brain networks and regions dynamically engage over the 19 time layers and 100 optimizations of the modularity maximization algorithm.

### Integration, recruitment, and flexibility within RSNs across the AD continuum

**Fig. 2:**
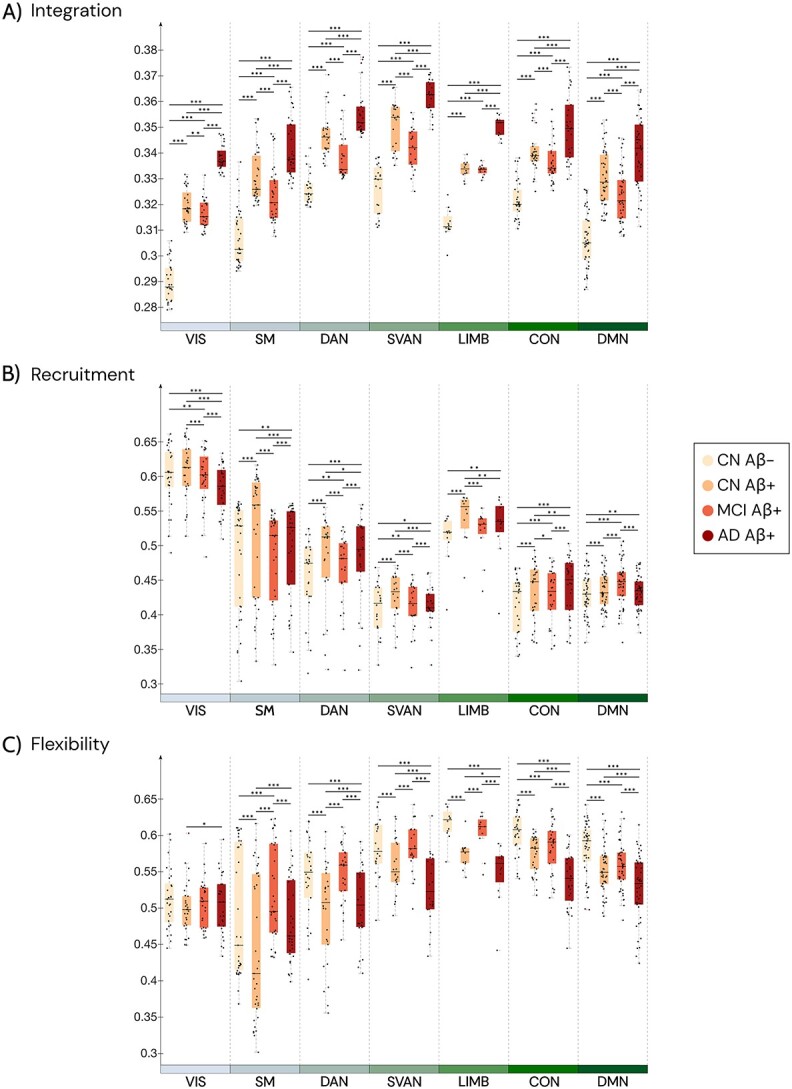
Visualization of our dynamic multilayer FC results for RSNs across different groups. Visualization of summary statistics for RSN-specific (A) integration, (B) recruitment, and (C) flexibility across the 19 time windows. The center black lines represent the median. Statistical analyses were performed while adjusting for sex and age and correcting for multiple comparisons using FDR, and significance levels are denoted as follows: ^*^P < 0.05, ^**^P < 0.01, ^***^P < 0.001. CON—control network; DMN—default mode network; DAN—dorsal attention network; SVAN—salience ventral attention network; LIMB—limbic network; SM—somatomotor; VIS—visual networks.

We discovered alterations in the temporal dynamics of functional brain networks in the Aβ + groups, including an increase in integration within most of the RSNs (Fig. 2A), as well as an increased recruitment in LIMB, DMN, CON, and DAN (Fig. 2B) compared to CN Aβ−. Moreover, there was a decrease in flexibility primarily within the LIMB, DMN, CON networks in these groups (Fig. 2C). These alterations may reflect underlying abnormalities in information processing and network structure in individuals with amyloid pathology.

**Fig. 3:**
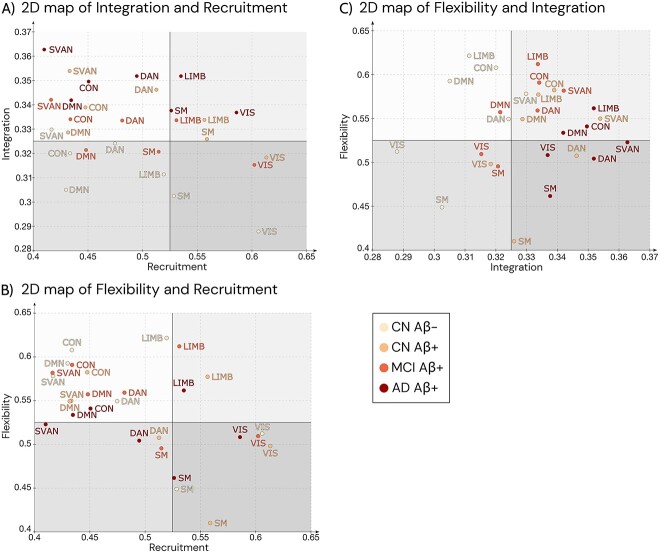
Relationship between each pair of dynamic multilayer measures in the AD continuum. Relationship between pairs of dynamic multilayer FC measures in terms of the RSN-specific median values: (A) recruitment-integration, (B) recruitment-flexibility, and (C) integration-flexibility. CON—control network; DMN—default mode network; DAN—dorsal attention network; SVAN—salience ventral attention network; LIMB—limbic network; SM—somatomotor; VIS—visual networks.

To further summarize these specific network results, we mapped the relationship between each pair of three dynamic measures into a two-dimensional space (Fig. 3). These plots offer a cartographic representation that can help us identify which measures and RSNs change more across the AD continuum. The division of the quadrants along each axis corresponds to the midpoint of the measurement range for each respective measure.

All RSNs were consistently recruited as network communities across time windows, but the level of self-recruitment varies between networks. While some networks, like VIS, SM, and LIMB, have consistently high levels of recruitment; others, including SVAN, DAN, CON, and DMN, have uneven levels of recruitment across time windows (Fig. 3A and 3B). On the contrary, integration was in general lower in all RSNs, with a weaker integration coefficient in VIS, DMN, and SM networks (Fig. 3A and C). Similarly to the recruitment, in general there was high flexibility in all RSNs and more specifically a constant high flexibility in the LIMB, DMN, CON and SVAN (Fig. 3B and C). Overall, RSNs’ patterns are consistent with findings from older individuals without neurological diseases (Malagurski et al. 2020).

### Dynamic multilayer functional connectivity measures enhance the accuracy of participant classification

**Fig. 4:**
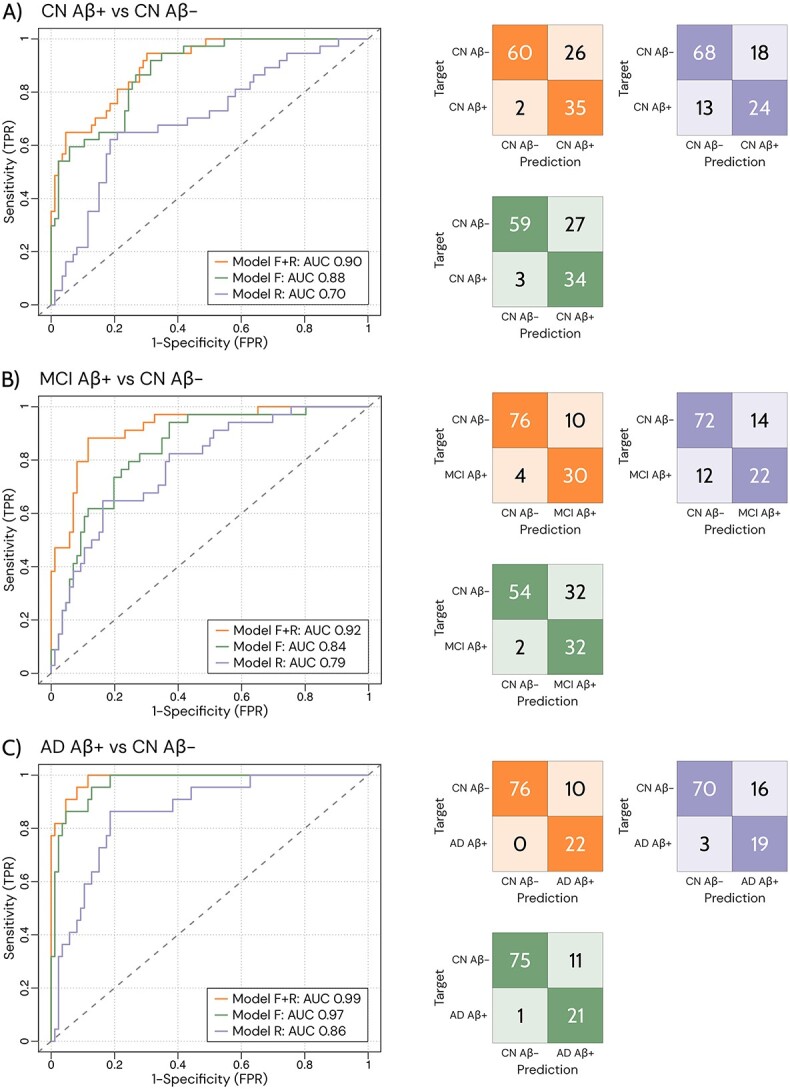
Group classification using dynamic multilayer FC measures and risk factors. ROC curves displaying the AUC scores (left) and confusion matrices (right) for each of the three classification models: CN Aβ + and CN Aβ- (A), MCI Aβ + and CN Aβ− (B), AD Aβ + and CN Aβ− (C) groups. Model F + R is the best model obtained by combining dynamic functional measures and risk factors, followed by model F that contains only the best combination of dynamic functional measures and finally model R that includes only the risk factors.

The performance of the 3 models for the group comparisons is shown in Fig. 4. The models using a combination of dynamic functional measures (Models F) are shown in green, while those using only risk factors (Models R) are shown in purple. The results show that Models F can categorize participants more accurately than Models R in all classifications (Fig. 4A–C). The performance is further improved by using a combination of our dynamic functional measures and selected risk factors (Models F + R) in all classifications, as shown by the models in orange (Fig. 4).

The best model that distinguished CN Aβ− from CN Aβ + (Fig. 4A, Model F + R), with an area under the curve (AUC) of 0.90, consisted of integration of VIS, the flexibility of CON, LIMB and SVAN, the recruitment of the SM, DMN and SVAN, as well as APOE ϵ4 and age. The AUC of model F + R was statistically different (*P* < 0.001, Supplementary Fig. 3A) than Model F (AUC of 0.88) and Model R (AUC of 0.71). In the discrimination between CN Aβ− and MCI Aβ + an AUC of 0.92 was obtained with measures of integration in VIS, LIMB and DAN, together with APOE ϵ4 and age (Fig. 4B, Model F + R). Model F + R also showed statistically significant (*P* < 0.001, Supplementary Fig. 3B) improvement over Model F (AUC of 0.84) and Model R (AUC of 0.79).

Finally, for the classification of CN Aβ− and AD Aβ + the best model had an AUC of 0.99 (Fig. 4C, Model F + R), and consisted of the integration in the VIS network and flexibility in the CON network together with APOE ϵ4 and age. Model F + R also outperformed Model F (AUC of 0.97) and Model R (AUC of 0.86) in terms of predictive accuracy (*P* < 0.001, Supplementary Fig. 3C).

### Measures of dynamic multilayer functional connectivity explain a large proportion of the variation in both cognitive function and AD pathology

To assess the relationship between our functional measures with cognition and pathology we employed partial least squares regression analyses in all the Aβ + groups together, while controlling for covariates.

**Fig. 5:**
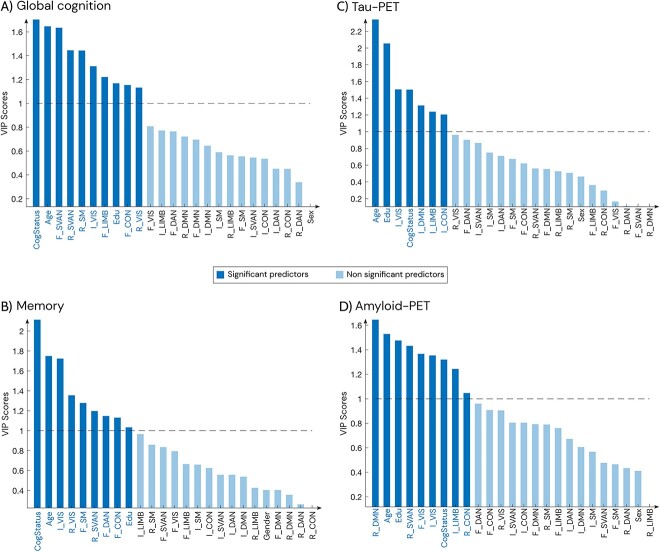
PLS regression analysis to assess the relationship of our dynamic multilayer FC measures with cognition and AD pathology. VIP scores determine significant predictors in the PLS model, which are highlighted in darker blue (VIP > 1), for global cognition (A), memory (B), tau-PET (C), and amyloid-PET (D). In light blue we show the non-significant predictors (VIP <= 1). F—flexibility; R—recruitment; I—integration; CogStatus—cognitive status; Edu—education. All PLS models were performed with all the Aβ positive subjects.

We found that flexibility in the SVAN, LIMB, and CON, recruitment in the SVAN, SM, and VIS, integration in VIS, as well as the cognitive status, age, and education were significant predictors of global cognition (Fig. 5A), explaining 30% of its variance. For memory, significant predictors included cognitive status, age, education, integration in VIS, recruitment in VIS and SVAN, and flexibility in SM, DAN, and CON, as shown in Fig. 5(B), which together explained 55% of variance. As can be observed in Fig. 5(C), we found that in addition to age, education, and cognitive status, integration in several networks such as the VIS, DMN, LIMB, and CON explained 14% of the variance in tau pathology. Finally, a combination of recruitment in DMN, SVAN, and CON, flexibility in VIS, and integration in VIS and LIMB, together with age, education, and cognitive impairment, as shown in Fig. 5(D), were significant predictors of amyloid pathology, explaining a total of 14% of variance.

The variance explained by each of the components or LVs for all PLS models can be found at Supplementary Fig. 4.

## Discussion

There is a need for non-invasive markers for the early diagnosis of AD that can also help characterizing different stages of disease progression. Although the development of plasma biomarkers has partly addressed this need (Hansson et al. 2022; Zetterberg and Schott 2022), this approach is still limited by the fact that it does not offer spatial information regarding which brain regions are affected, only showing significant abnormalities once the levels of blood biomarkers have surpassed a certain threshold at the whole-brain level. Therefore, imaging biomarkers that can perform an effective diagnosis of different disease stages are still needed to offer insights regarding which brain areas are most affected and their association with cognitive impairment. In this study, we assessed dynamic multilayer functional connectivity from preclinical to clinical AD stages. In doing so, we found significant connectivity alterations across the AD continuum that are useful to discriminate earlier and later disease stages and that are closely related to cognition as well as to amyloid and tau pathology. Thus, these sensitive and dynamic measures should be considered by future studies aiming to identify different AD stages using non-invasive brain imaging techniques, especially preclinical stages.

Our findings in cognitively normal individuals without amyloid pathology, the CN Aβ− group, are consistent with previous studies indicating that RSNs are consistently recruited as network communities across time windows although with observed differences in the strength of self-recruitment among networks (Mattar et al. 2015; Malagurski et al. 2020). Particularly, for the whole rs-fMRI scan, the VIS, SM, and LIMB networks featured high levels of recruitment. In contrast, the SVAN, DAN, CON, and DMN displayed uneven levels of recruitment during different time windows. Additionally, our findings suggest a general trend of high flexibility in the CN Aβ− group in all RSNs, with the LIMB, DMN, CON, and SVAN consistently exhibiting high flexibility levels. On the contrary, integration in the CN Aβ− group is generally low in all RSNs, with a weaker integration coefficient in the VIS, DMN, and SM networks. Therefore, our results suggest that RSNs exhibit unique temporal dynamics. Some RSNs display more stable recruitment, indicating greater temporal coherence. Other RSNs exhibit low integration, suggesting a tendency for them to become more temporally segregated. Finally, some RSNs show greater local flexibility, making them more adaptable. This is in line with the adoption of a more energy-efficient strategy while at rest, which can be viewed as economical brain functioning (Mattar et al. 2015).

When looking into the alterations of our dynamic functional measures along the AD continuum, we observed that, in general, the flexibility was decreased in all the RSNs, with the exception of the SM network. This result concurs with previous research emphasizing the critical role of network flexibility in cognitive processing and the loss of this function in a variety of neurological disorders (Pedersen et al. 2018; Gifford et al. 2020; Yang et al. 2022). In addition, across the AD continuum, the dynamic integration coefficient was increased in all RSNs, and the dynamic recruitment coefficient was either increased or maintained in most RSNs, except for the VIS network, which showed a decrease in recruitment. These alterations indicate a decrease in temporal segregation and an increase in integration, leading to a shift in the balance between segregation and integration across the AD continuum towards a more random network organization. This is consistent with earlier findings that showed lower selectivity and decreased category-specific activation as people grow older, where lower selectivity reflects a decline in the functional specialization of different regions within the brain (Chan et al. 2017).

Overall, the findings from the logistic regression models support our initial hypothesis that a combination of dynamic multilayer FC measures (Models F in Fig. 4) is more accurate for discriminating patients in the AD continuum than using only risk factors (Models R in Fig. 4). This was the case for all classifications, including those that distinguished between the CN Aβ− group and the CN Aβ + groups. Furthermore, integrating our dynamic functional measures with certain risk factors (Models F + R in Fig. 4) considerably improved the performance of our classifications (*P* < 0.001, Supplementary Fig. 3). These findings underline the importance of dynamic functional measures in the evaluation of AD, particularly in identifying individuals in early stages, which has important implications for patient selection in clinical trials and interventions.

We also evaluated the clinical significance of the dynamic multilayer FC measures by examining their association with the extent of brain pathology using amyloid-PET and tau-PET measures as well as the scores of global cognitive and memory tests that are frequently employed in clinical settings to evaluate individuals with signs of AD. The dynamic functional measures were associated both with global amyloid and temporal tau burden, which is in line with prior findings revealing a correlation between network structure and amyloid and tau pathology (Sintini et al. 2021). Specifically, we observed that dynamic measures from the DMN, an RSN containing regions that show the earliest deposition of amyloid, were some of the most important predictors of tau and amyloid pathology (in amyloid the recruitment in DMN was the most important). Our measures were also related to cognition and memory. Flexibility and recruitment in SVAN, flexibility in the LIMB, CON as well as recruitment in SM and integration in VIS were found to be the main predictors of cognition after age and cognitive impairment, which is consistent with previous research demonstrating that regions that are more flexible are linked to the default mode, cognitive control, and executive networks (Harlalka et al. 2019). Similarly, integration, flexibility, and recruitment in cognitive and sensorial networks were associated with memory. This suggests that the dynamic properties of these RSNs play a role in memory processing.

Despite the value of our study in offering a promising way to study functional alterations over time across the AD continuum, there are some important methodological considerations that need to be acknowledged. First, we relied on cross-sectional imaging data, being thus unable to evaluate the potential predictive value of dynamic multilayer FC measures over the years and their longitudinal relationship with cognition and pathology. Furthermore, despite the utility of dFC, certain controversies persist and warrant consideration when interpreting results. These include issues related to eliminating or accounting for noise from fMRI time series, selecting suitable window lengths, and the possibility that dFC patterns might be influenced by the modulation of neural activity (Hutchison et al. 2013). To address such potential issues, we tried three different window lengths, which were chosen based on previous studies performing dynamic network analyses at the resting state (Leonardi and Van De Ville 2015). Despite the differences in window lengths, we observed that the changes along the AD continuum followed a consistent pattern with recruitment increases in addition to flexibility and integration decreases with longer windows.

Moreover, it is important to recognize that these findings are derived from a relatively small sample. Future research must confirm our results by replicating them in independent and larger samples to enhance the results’ reliability and generalizability. Such replication efforts could benefit from a clinical environment with broader inclusion criteria that permits the inclusion of younger patients. Additionally, in future studies, the incorporation of alternative measures not solely reliant on community detection, such as efficiency metrics for multiplex or multilayer brain networks, could significantly contribute to comprehensive analyses. Another important consideration is the brain atlas used to define the nodes in the network analysis since it has been shown to have a significant impact on the results (Fornito et al. 2016) and it is important to consider this when interpreting them. We based our nodes on a functional atlas that consists of seven RSNs (Thomas Yeo et al. 2011; Schaefer et al. 2018), which has been frequently employed in previous research using a similar methodology. Finally, the parameters of the multilayer community detection algorithm play an important role in determining the number, size, and similarity of communities across time windows. We assessed the similarity across time windows for various omegas and gammas in the group-level outcomes. For our study, we used the default gamma = 1 and omega = 0.5 similarly to previous research (Puxeddu et al. 2020) since it provides a good trade-off between distinct and common modular structures in successive time windows.

## Conclusion

To conclude, in this study we demonstrate that alterations in the temporal dynamics of functional brain networks, as measured by resting-state functional MRI, change across the AD continuum. In the Aß + groups, we discovered greater recruitment in certain networks and increased integration in the majority of the RSNs, including the earliest stages of the disease in the preclinical participants. Moreover, the limbic, default mode, control, and attention networks showed a decline in flexibility. We were able to categorize participants more accurately by combining functional measures than we could have done with just risk factors, demonstrating the potential of dynamic multilayer FC measurements to increase participant classification accuracy, especially in the early stages. Finally, additional support for the potential of some of our multilayer dynamic functional measures as functional biomarkers for AD was provided by the fact that they also significantly correlated with cognition and AD pathology.

## Supplementary Material

Supplementary_Figure1_bhad542

Supplementary_Figure2_bhad542

Supplementary_Figure3_bhad542

Supplementary_Figure4_bhad542

updated_Supplementary_Materials_bhad542
