# Supplementary figures and images for: Dynamic multilayer functional connectivity detects preclinical and clinical Alzheimer’s disease
Source: Cereb Cortex. 2024 Jan 11;34(2):bhad542. doi: 10.1093/cercor/bhad542 (PMC10839846; doi:10.1093/cercor/bhad542)

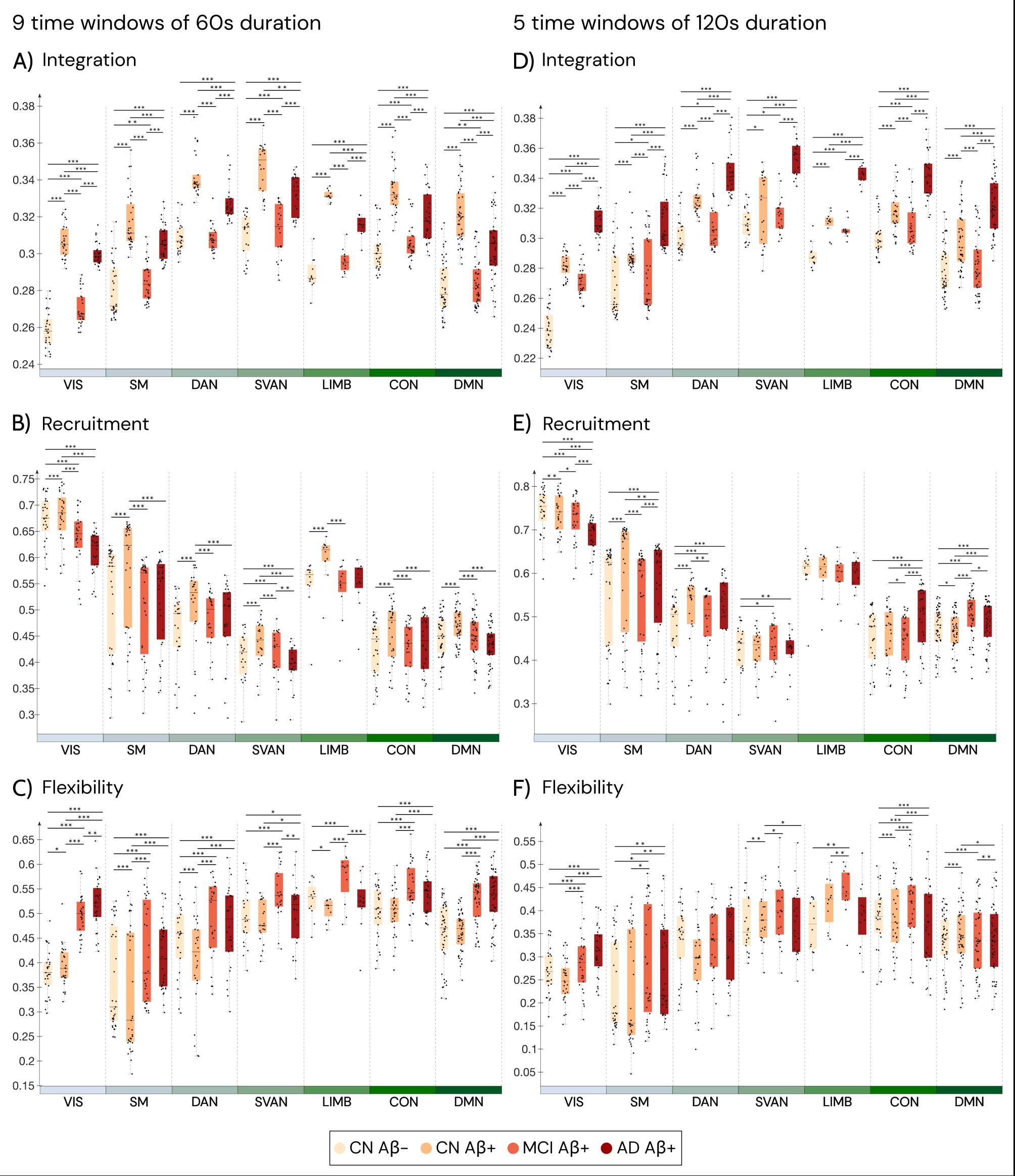

Supplement: Supplementary_Figure1_bhad542 [file supplementary_figure1_bhad542.jpeg]

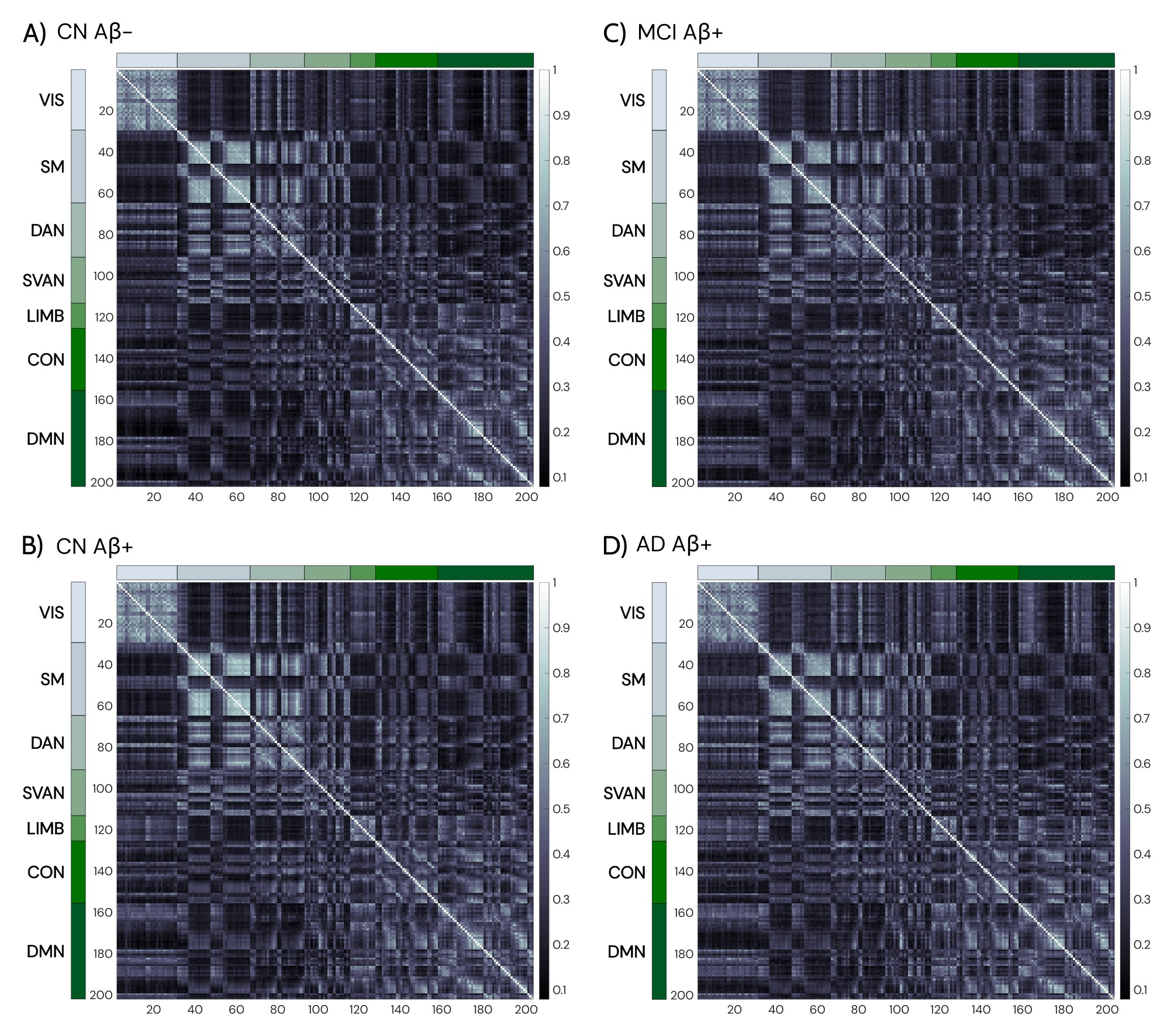

Supplement: Supplementary_Figure2_bhad542 [file supplementary_figure2_bhad542.jpeg]

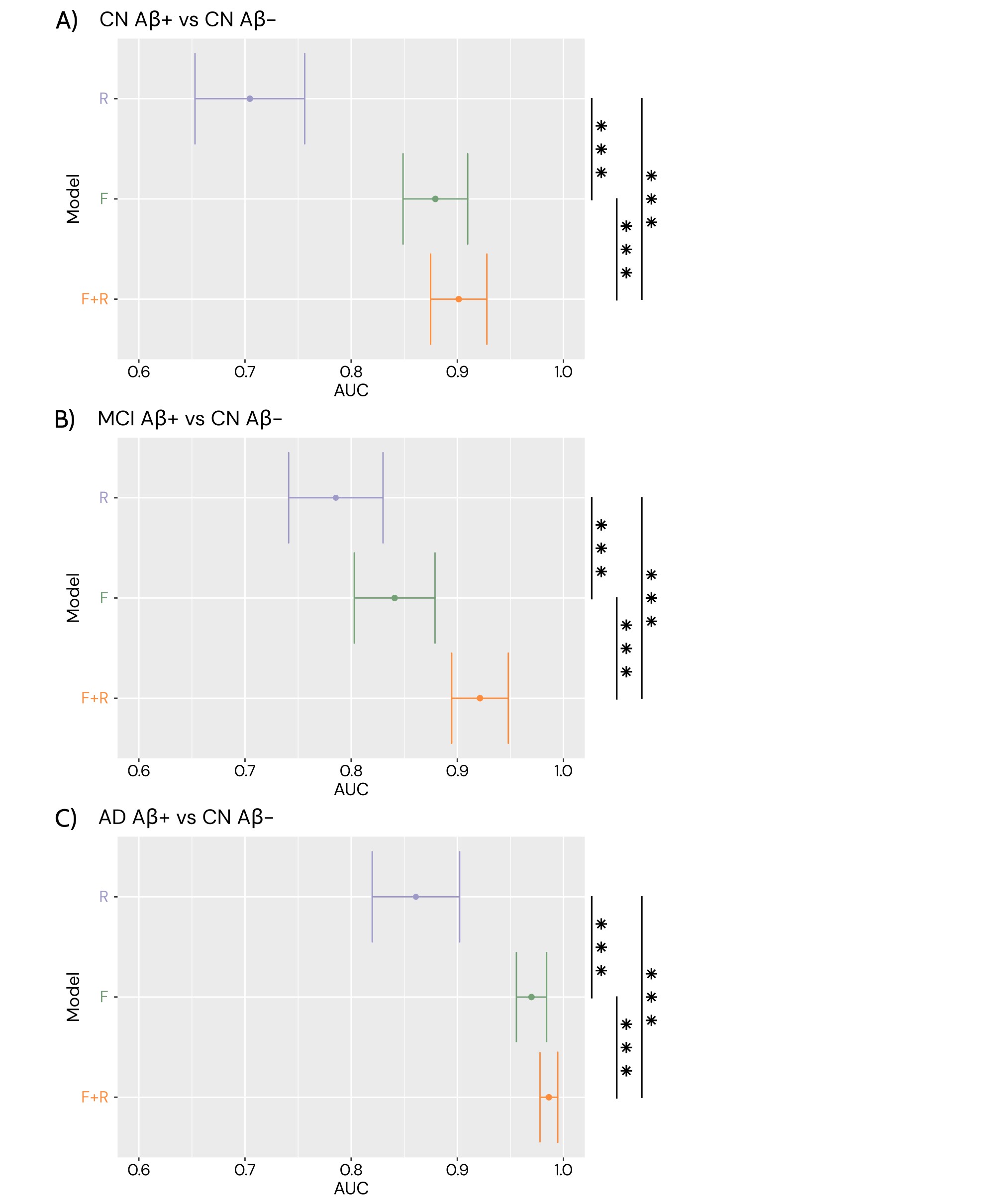

Supplement: Supplementary_Figure3_bhad542 [file supplementary_figure3_bhad542.jpeg]

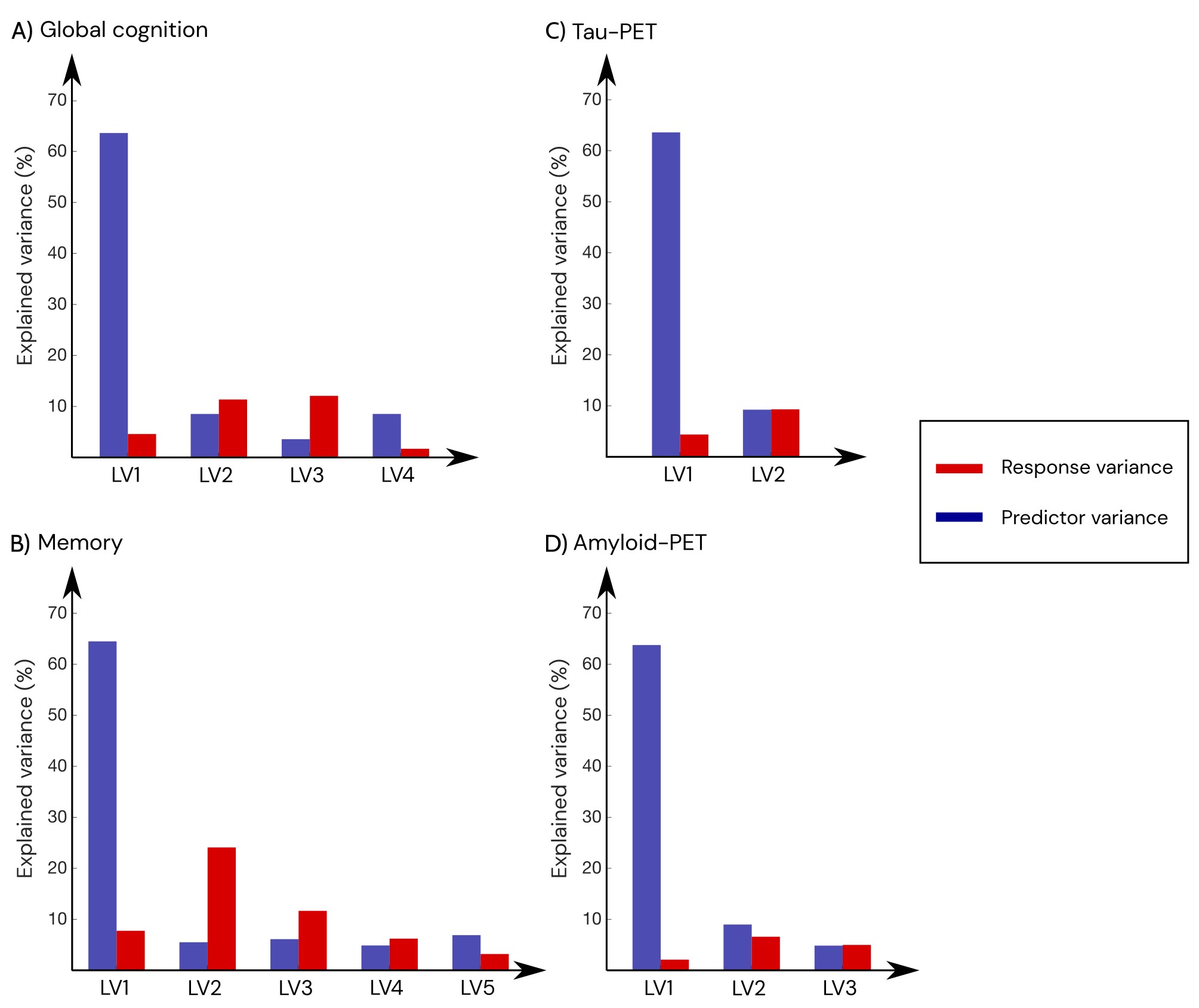

Supplement: Supplementary_Figure4_bhad542 [file supplementary_figure4_bhad542.jpeg]
