# Supplementary material for: Dynamic multilayer functional connectivity detects preclinical and clinical Alzheimer’s disease
Source: Cereb Cortex. 2024 Jan 11;34(2):bhad542. doi: 10.1093/cercor/bhad542 (PMC10839846; doi:10.1093/cercor/bhad542)
Supplement: updated_Supplementary_Materials_bhad542 [file updated_supplementary_materials_bhad542.docx]

**Supplementary Figures**

**Supplementary Figure 1. Visualization and summary of our dynamic multilayer functional connectivity results for RSNs and different groups using different window sizes.**


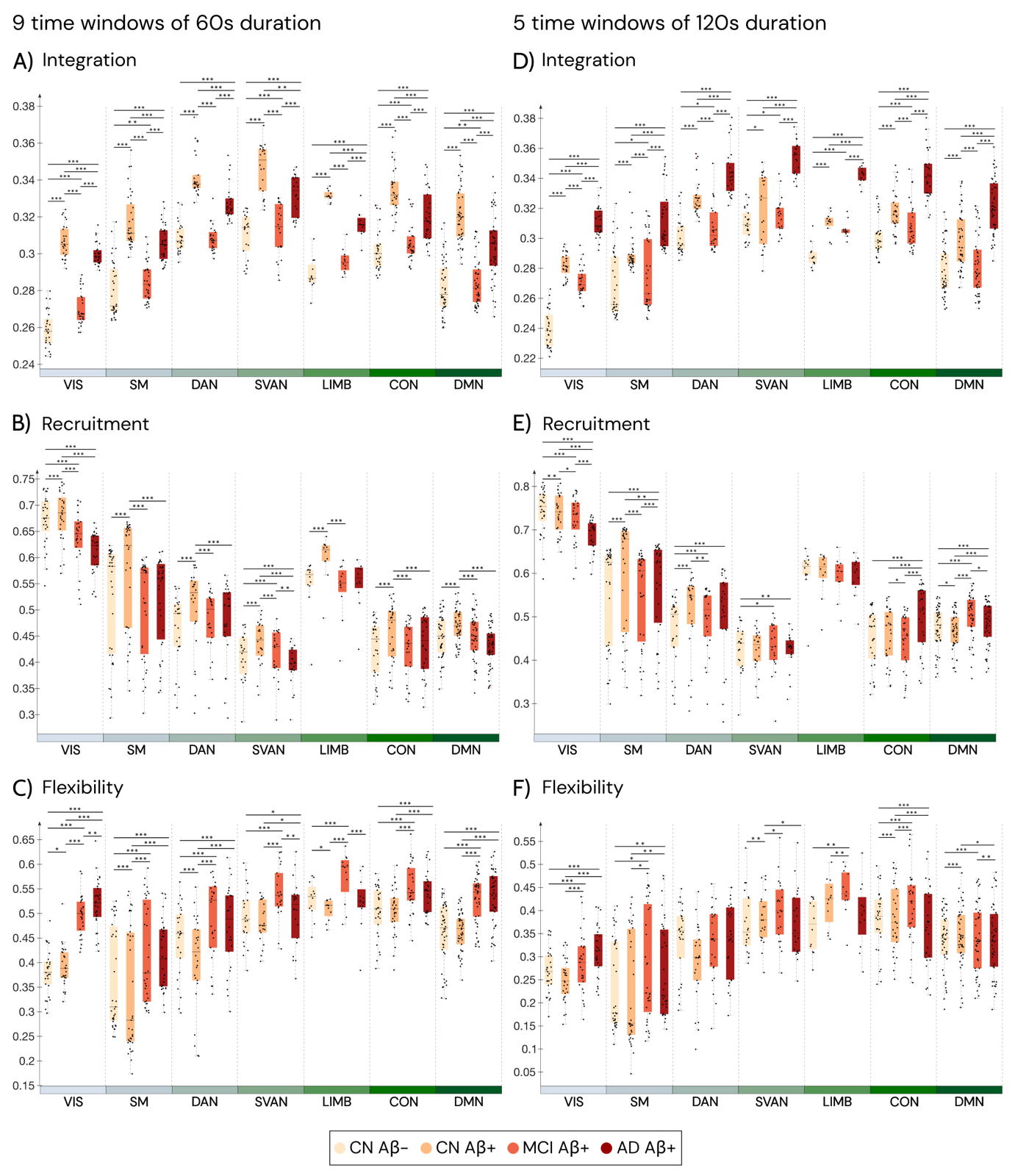


Visualization of summary statistics for resting-state network-specific a) integration, b) recruitment, and c) flexibility across the 9 time windows of 60s duration and d) integration, e) recruitment, and f) flexibility across the 5 time windows of 120s duration. The center black lines represent the median. Statistical analyses were performed while adjusting for sex and age and correcting for multiple comparisons using FDR, and significance levels are denoted as follows: *p < 0.05, **p < 0.01, ***p < 0.001.

CON – control network; DMN – default mode network; DAN – dorsal attention network; SVAN – salience ventral attention network; LIMB – limbic network; SM- somatomotor; VIS – visual networks.

**Supplementary Figure 2. Module Allegiance matrices.**

**
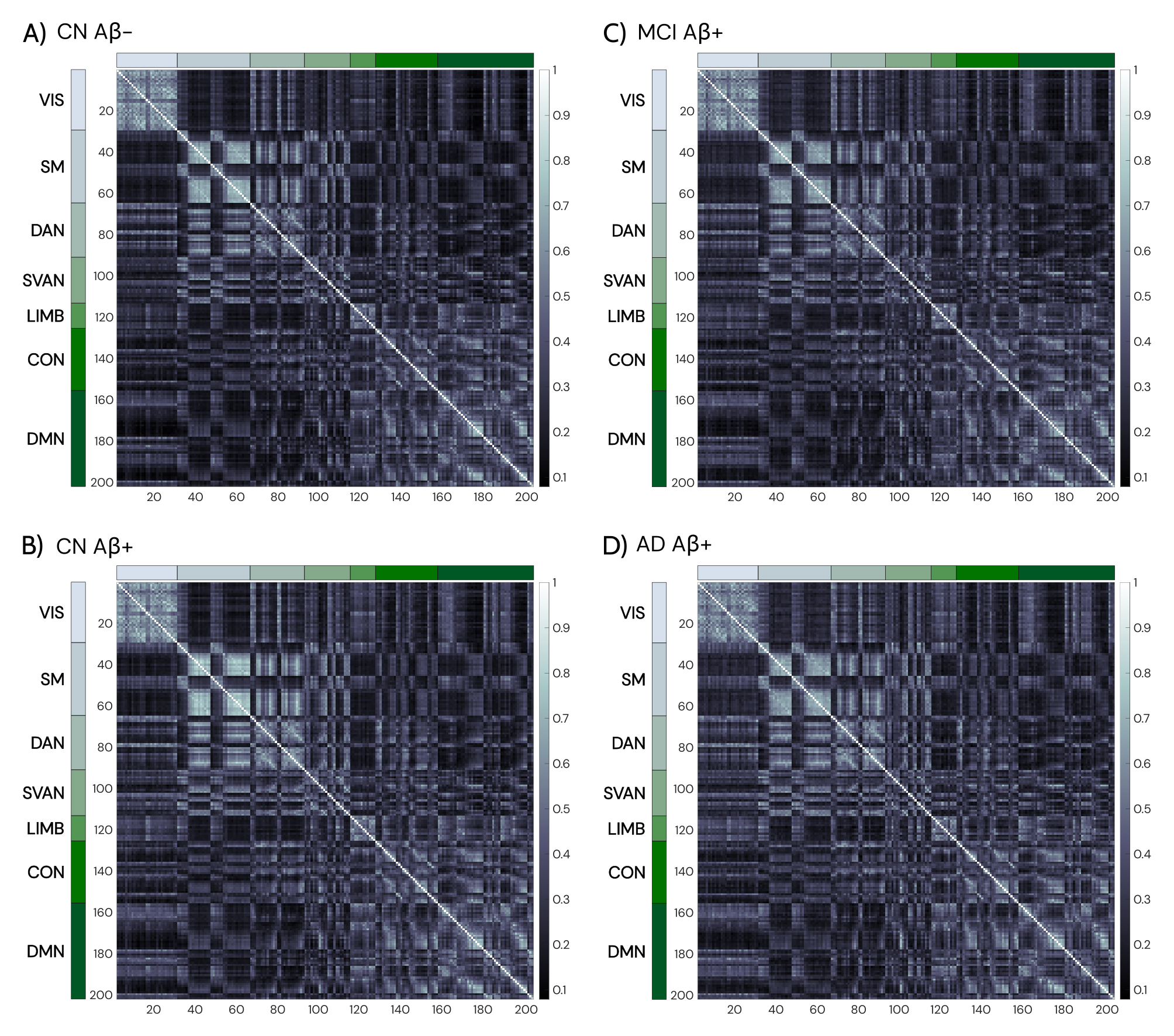
**

Module allegiance matrices for CN Aβ- (a), CN Aβ+ (b), MCI Aβ+ (c), and AD Aβ+ (d) groups obtained from the calculation of multilayer communities in the 19 layers using 100 optimizations.

CON – control network; DMN – default mode network; DAN – dorsal attention network; SVAN – salience ventral attention network; LIMB – limbic network; SM- somatomotor; VIS – visual networks.

**Supplementary Figure 3. Models’ comparison.**


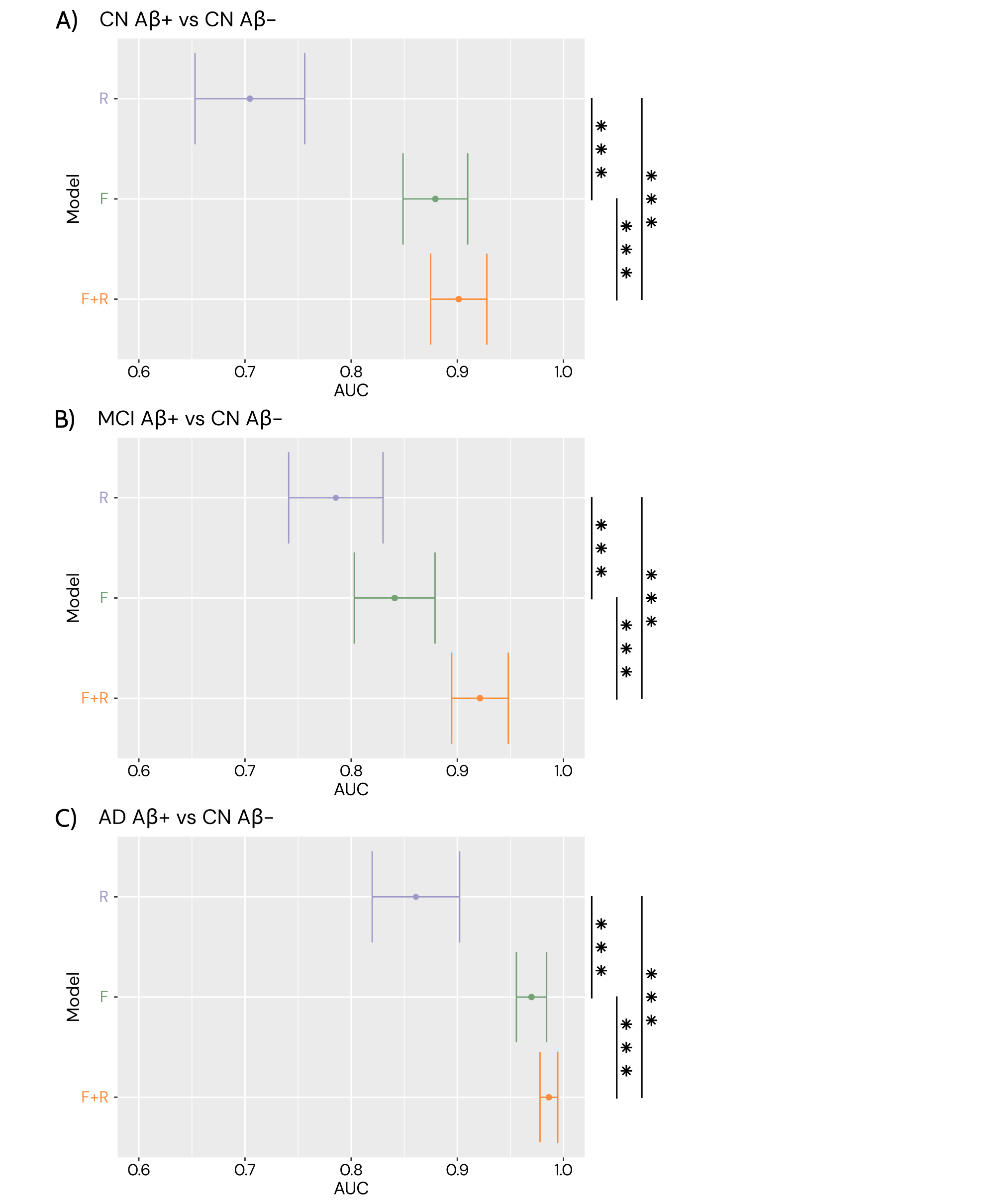


Comparison of the three models for each classification: CN Aβ+ and CN Aβ- (A), MCI Aβ+ and CN Aβ- (B), AD Aβ+ and CN Aβ- (C) groups. (A) Model R has an AUC of 0.70 (std 0.052), model F an AUC of 0.88 (std 0.031), and model F+R an AUC of 0.90 (std 0.028). (B) Model R has an AUC of 0.79 (std 0.045), model F an AUC of 0.84 (std 0.037), and model F+R an AUC of 0.92 (std 0.026). (C) Model R has an AUC of 0.86 (std 0.039), model F an AUC of 0.97 (std 0.015), and model F+R an AUC of 0.99 (std 0.008). In all classifications, all models are statistically different (<0.001). The statistical difference between models was evaluated using Kruskal-Wallis tests applied to the 1000 AUC replicates.

**Supplementary Figure 4. Explained variance of the individual components for the different PLS models.**


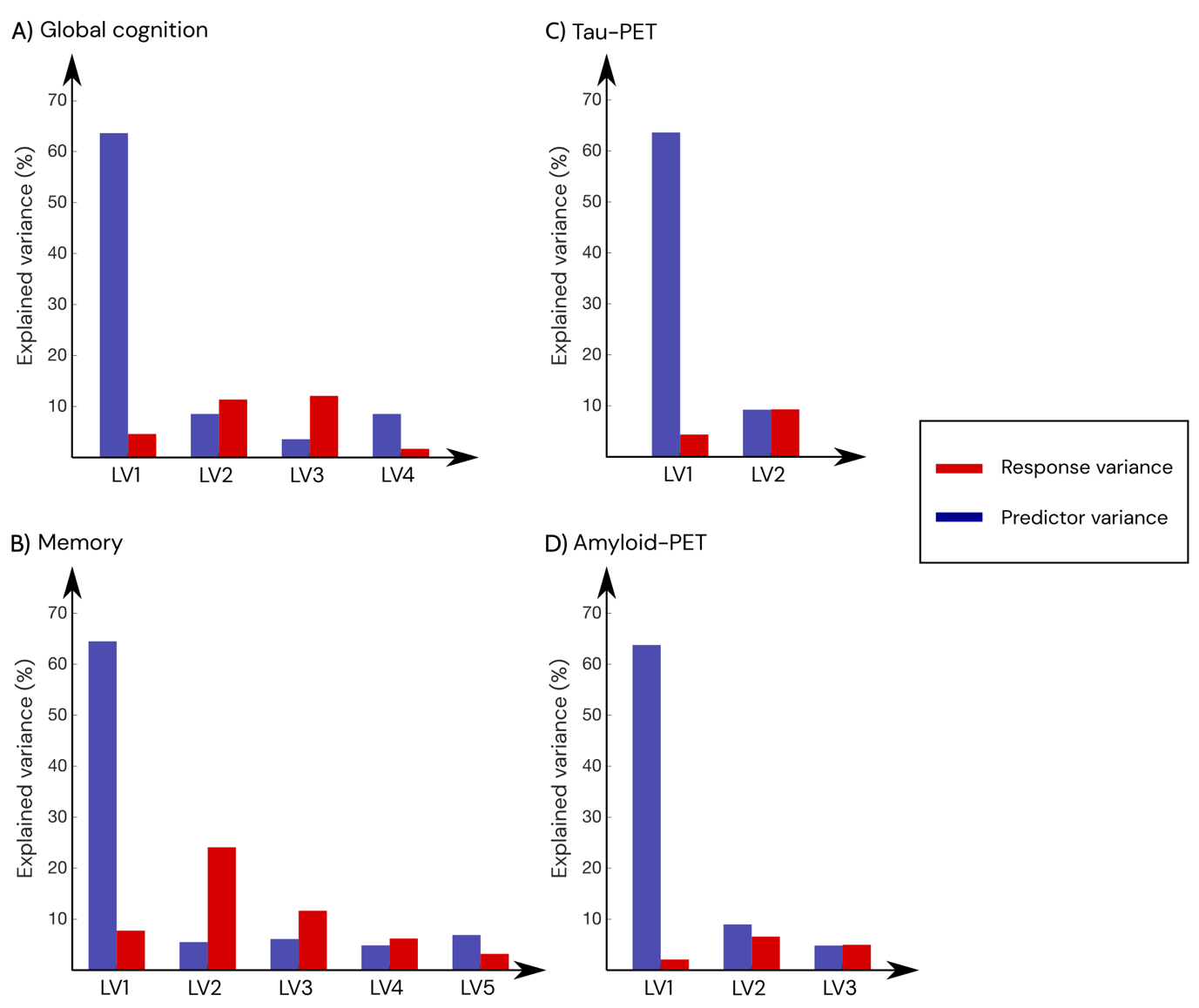


Explained predictor (in blue) and response (in red) variance by each latent variable (LV) in global cognition (a), memory (b), tau-PET (c), and amyloid-PET (d) PLS models.
